# Typology and formation of the arterial circle at the base of the brain in neonatal Saanen goats

**DOI:** 10.29374/2527-2179.bjvm005825

**Published:** 2025-12-12

**Authors:** Edmilla Pastor Marquez, Marcelo Salvador Gomes, Anieli Vidal Stocco, Marcelo Soares Antunes, Paulo Oldemar Scherer, Marcelo Abidu-Figueiredo

**Affiliations:** 1 Instituto de Veterinária, Universidade Federal Rural do Rio de Janeiro, Seropédica, RJ, Brazil.; 2 Departamento de Anatomia Animal e Humana, Instituto de Ciências Biológicas e da Saúde, Universidade Federal Rural do Rio de Janeiro, Seropédica RJ, Brazil.; 3 Programa de Pós-Graduação em Medicina Veterinária, Departamento de Medicina e Cirurgia Veterinária, Universidade Federal Rural do Rio de Janeiro, Seropédica, RJ, Brazil.

**Keywords:** measurements, central nervous system, vascularization, medidas, sistema nervoso central, vascularização

## Abstract

Diseases affecting the central nervous system of domestic animals account for a substantial proportion of the conditions encountered in clinical and surgical practice involving production animals. While the cerebral vascular anatomy is well characterized in humans; however, detailed information remains limited for many mammalian species, particularly regarding the origin of the basilar artery and the contribution of the vertebral artery. The study of the arteria distribution responsible for cerebral irrigation in domestic animals and species used as experimental models, from a phylogenetic perspective, remains relevant in biomedical research due to the high variability in vascular arrangements among species. This study aimed to describe the typology and formation of the arterial circle at the base of the brain in neonatal Saanen goats. Eleven heads from cadavers approximately one month of age (5 males and 6 females) were dissected, after fixation in 10% formaldehyde, followed by arterial injection with colored Petrolátex S-65. The mean and standard deviation of the basilar artery length was 2.5 ± 0.30 cm in females and 2.6 ± 0.22 cm in males. In all specimens, the basilar artery originated from the anastomosis of the right and left vertebral arteries, giving rise to branches supplying the medulla oblongata, the caudal cerebellar arteries, pontine branches, terminal branches, and rostral cerebellar arteries. The arteries at the base of the brain were supplied by both the carotid and vertebrobasilar systems. The encephalic vascular pattern showed a tendency toward type II. These findings are fundamental for understanding the cerebral vascularization of the species and its clinical-surgical applications.

## Resumo

As doenças que acometem o sistema nervoso central de animais domésticos representam parcela significativa das afecções observadas na prática clínico-cirúrgica em animais de produção. A anatomia vascular cerebral é bem conhecida em humanos, porém há escassez de informações detalhadas para muitas espécies de mamíferos, especialmente quanto à origem da artéria basilar e à participação da artéria vertebral. O estudo da distribuição das artérias responsáveis pela irrigação encefálica em animais domésticos e em espécies utilizadas como modelos experimentais, sob a ótica filogenética, segue sendo relevante na pesquisa biomédica, devido à grande variabilidade nos arranjos vasculares entre as espécies. Este trabalho teve como objetivo descrever a tipologia e formação do circuito arterial da base do encéfalo em caprinos recém-nascidos da raça Saanen. Foram dissecadas 11 cabeças de cadáveres com cerca de um mês de idade (5 machos e 6 fêmeas), previamente fixadas em formaldeído a 10%, com posterior preenchimento arterial com Petrolátex S-65 corado. A média e o desvio padrão do comprimento da artéria basilar foram de 2,5 ± 0,30 cm nas fêmeas e 2,6 ± 0,22 cm nos machos. Em todos os caprinos, a artéria basilar originou-se da anastomose entre as artérias vertebrais direita e esquerda, emitindo ramos para o bulbo, artérias cerebelares caudais, ramos para a ponte, ramos terminais e artérias cerebelares rostrais. As artérias da base encéfalo dependiam dos sistemas carótico e vertebrobasilar. O padrão vascular encefálico apresentou tendência ao tipo II. Esses achados são fundamentais para a compreensão da vascularização cerebral da espécie e suas aplicações clínico-cirúrgicas.

## Introduction

Diseases affecting the central nervous system of domestic and wild animals account for a considerable proportion of the conditions encountered in the clinical and surgical practice of production animals (Barbosa et al., 2024) and may result in a reduced blood supply and consequent ischemic injury (Meneses, 2011). Surgical interventions in this region require precise anatomical knowledge, not only of the normal morphology but also of the structural variations that may occur.

Cerebral vascularization in humans is well established, however, detailed information remains limited for many mammalian and non-mammalian species, particularly regarding the origin of the basilar artery and the contribution of the vertebral artery (Salaud et al., 2024). Although the internal carotid artery consistently supplies the brain in all vertebrates (Rahmat & Gilland, 2014), the involvement of the vertebral artery and the origin of the basilar artery vary among vertebrates groups and even within certain species. Therefore, research aimed at improving the understanding of this anatomy can enhance the reliability of animal models used in radiological and surgical studies and contribute to a better understanding of anatomical variations in both humans and in experimental animals.

According to the literature, the role of the vertebral artery in supplying the encephalic arteries varies considerably among vertebrates. In anamniotes (fish and amphibians), the basilar artery may be single or paired and arises from the caudal branches of the internal carotid artery (Rahmat & Gilland, 2014). Amniotes, which include diverse taxa such as turtles, lizards, and birds, exhibit rostral-to-caudal cerebral blood flow through the carotid-basilar arterial system, whereas mammals demonstrate caudal-to-rostral blood flow through the vertebro-basilar system (Rahmat & Gilland, 2014). Certain mammals, such as ruminants, possess a carotid rete mirabile supplied by branches of the external carotid artery, including the maxillary artery (Gillilan, 1974; Karasawa, et al., 1997).

In a phylogenetic study on cerebral arteries, Vriese (1904) classified the encephalic blood supply of vertebrates into three types: Type I: in which the brain is supplied almost exclusively by the internal carotid arteries; Type II: in which the brain is partially supplied by both the carotid and vertebro-basilar systems, with either an equal distribution between the two systems or a predominance of one of them; and Type III: in which the brain is supplied almost exclusively through the vertebro-basilar system.

The study of the arterial distribution responsible for cerebral blood supply in domestic animals and those used as experimental models, from a phylogenetic perspective, remains an important topic in biomedical research due to the high variability in arterial arrangements.

Throughout the evolutionary development of the central nervous system, continuous modifications in the configuration of the vessels responsible for its vascularization have been observed (Lima et al., 2006). The aim of the present study was to establish the typology and formation of the arterial circle at the base of the brain in neonatal Saanen goats.

## Material and methods

Anatomical dissection were performed on 11 heads from neonatal Saanen goat cadavers, approximately one month of age (5 males and 6 females). The heads were sourced from the collection of the Department of Animal and Human Anatomy at the Federal Rural University of Rio de Janeiro. The carcasses had been previously fixed in 10% formaldehyde solution, following standard anatomical procedures. Subsequently, the arterial system was injected with an aqueous solution (1:1 dilution) of Petrolátex S-65 (Refinaria Duque de Caxias – REDUC, Petrobrás, Duque de Caxias-RJ) mixed with dye (Suvinil Xadrez®).

Afterwards, the cranial vaults were removed and openings were made in the meninges. The specimens were then immersed in a 30% formalin solution for five days to complete brain fixation and the coagulation of the latex. Standard surgical instruments and dental forceps were used to open the the skulls, remove the brains, and dissect the vessels at the brain base.

The vessels at the base of the brain were dissected, photographed for documentation, and identified according to the International Committee on Veterinary Gross Anatomical Nomenclature (2017). The mean and standard deviation of the basilar artery length was calculated. These measurements were compared between sides and sexes using the unpaired Student’s t-test, considering p < 0.05 as statistically significant. All data processing were performed using GraphPad Prism 5 software.

## Results

### Basilar artery

**Figure 1:**
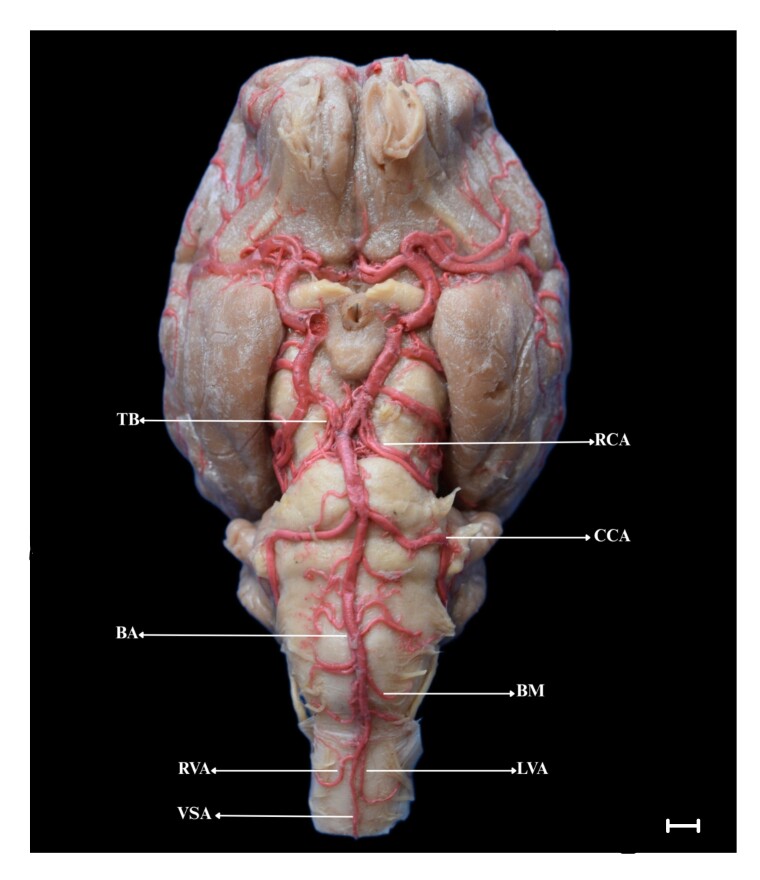
Digital photomacroscopy of the ventral view of the goat brain. Scale bar: 1 cm.

*Note:* Ventral spinal artery (VSA), right and left vertebral arteries (RVA and LVA), basilar artery (BA), branches to the medulla oblongata (BM), caudal cerebellar artery (CCA), rostral cerebellar artery (RCA), terminal branch of the basilar artery (TB).

In all examined specimens, both males and females, the right and left vertebral arteries were identified at the junction of the bulb and spinal cord, where they anastomosed and continued rostrally as the basilar artery and caudally as the ventral spinal artery (Figure 1). The mean length of the basilar artery, with standard deviation, was 2.6 ± 0.22 cm in males and 2.5 ± 0.30 cm in females (p = 0.5305).

In 4 (80%) males, the basilar artery was formed by the union of the right and left vertebral arteries with the contribution of the ventral spinal artery (Figure 1). In only 1 (20%) male, the ventral spinal artery did not participate in its formation. The basilar artery showed a sinuous course in 2 (40%) of the samples and a straight course in 3 (60%). This vessel was single in 4 (80%) of the samples and double in 1 (20%). When double, the basilar artery exhibited this pattern exclusively in the pontine region. Up to the beginning of the pons, it traveled as a single vessel, where it then bifurcated, becoming a double vessel.

The first branches originating from the basilar artery were directed to the bulb, varying from 7.6 ± 1.5 cm on the right side and 7.0 ± 1.5 cm on the left side. Subsequently, at the level of the bulbo-pontine sulcus, it gave off the caudal cerebellar artery, which had no secondary branches in any specimen. This branch had symmetrical positioning between sides in 3 (60%) of the samples and asymmetry in 2 (40%). Among the asymmetric cases, in 1 (50%) of the data, this branch originated more caudally on the left side and more rostrally on the right side. In the other 1 (50%), the origin was more caudal on the right side and more rostral on the left. Next, it emitted branches to the pons, which varied from 3.2 ± 1.3 cm on the right side and 3.0 ± 1.2 cm on the left side.

Following the bifurcation of the basilar artery giving rise to its terminal branches, the rostral cerebellar artery was emitted, which showed branching in 3 (60%) of the samples and remained as a single vessel in 2 (40%). Among the branching cases, branches were present on both sides in 1 (34%) of the samples and only on the left side in 2 (66%). No exclusive branching on the right side was observed. This branch showed 100% symmetrical emergence between sides.

In all females, the basilar artery was formed by the union of the right and left vertebral arteries with the contribution of the ventral spinal artery. The basilar artery exhibited a straight course in all specimens (Figure 1).

This vessel was single in 3 (50%) specimens and double in 3 (50%). When double, the basilar artery exhibited this pattern exclusively in the region of the pons. Up to the beginning of the pons, it followed a single course, at which point it bifurcated, becoming a double vessel.

The first branches of the basilar artery were directed to the bulb, measuring 6.5 ± 1.6 cm on the right side and 6.5 ± 0.55 cm on the left side (Figure 1). Subsequently, at the level of the bulbo-pontine sulcus, it gave off the caudal cerebellar artery, which showed symmetrical positioning between sides in 4 (66%) of the samples and asymmetry in 2 (34%). In the asymmetric cases, in both specimens (100%), the branch on the left side originated more caudally, while the branch on the right side originated more rostrally. In one specimen (16%), the caudal cerebellar artery gave off two branches on each side, showing both numerical and positional symmetry. Next, it emitted branches to the pons, ranging from 4.2 ± 1.2 cm on the right side and 4.3 ± 1.9 cm on the left side. After the bifurcation of the basilar artery into its terminal branches, the rostral cerebellar artery arose. This artery showed symmetrical positioning between sides in 83.3% (5) of the individuals and asymmetry in 16.7% (1), in which the branch on the left side originated more caudally than on the right. The rostral cerebellar artery did not show branching in 83.3% (5) of the specimens. In the only case of branching, numerical symmetry was observed between both sides.

### Formation of the arterial circle at the base of the encephalon

**Figure 2:**
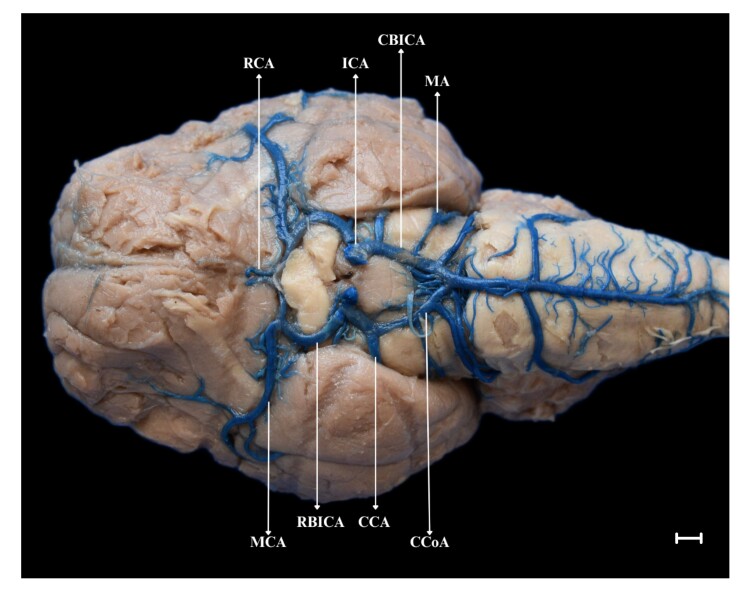
Digital photomacroscopy of the ventral view of the goat brain. Scale bar: 1 cm.

*Note:* Internal carotid artery (ICA), rostral branch of ICA (RBICA), rostral cerebral artery (RCA), middle cerebral artery (MCA), caudal branch of ICA (CBICA), caudal cerebral artery (CCA), mesencephalic artery (MA), and caudal communicating artery (CCoA).

The general arrangement of the arteries at the base of the encephalon, in both sexes, depends on the carotid and vertebrobasilar systems on both sides, formed by blood vessels whose branches vary in number and disposition. In goats, the arterial circle of the encephalon forms an irregular geometric shape, surrounding the optic chiasm, tuber cinereum, and hypophysis rostrally, and the mammillary body and the interpeduncular fossa caudally. Thus, the arterial circle extends from the medial portion of the cerebral peduncle to the rostral margin of the optic chiasm (Figure 2).

The arterial pattern observed at the base of the encephalon in these animals corresponds to Type II of the classification proposed by by Vriese (1904). In this pattern, the arteries of the carotid system display a rostrocaudal flow direction, whereas those of the vertebrobasilar system exhibit a caudorostral flow direction. The anastomosis between the caudal communicating arteries and the terminal branches of the basilar artery represents the convergence point of these two flow currents**.**

The vessels delimiting the arterial circle were: rostrally, the rostral branch of the internal carotid artery, the rostral cerebral arteries, and the rostral communicating artery; and caudally, the caudal branches of the internal carotid artery, the caudal communicating artery, and the terminal branches of the basilar artery.

The caudal branches of the internal carotid artery of the encephalon, on both sides, originate from the homonymous arteries, course caudally, and terminate shortly after the emergence of the mesencephalic arteries, in the ventral region of the mammillary body. From this point, these arteries continue caudally as the caudal communicating arteries, which subsequently form an anastomosis on the ventral surface of the cerebral peduncle with the corresponding terminal branches of the basilar artery. Arising from the caudal portion of the internal carotid artery are the caudal cerebral arteries and the mesencephalic arteries.

In females, the caudal cerebral artery originated from the caudal branch of the internal carotid artery in 5 (83%) of the examined female specimens, whereas in 1 (17%), it originated from the anastomosis between the rostral and caudal branches of the internal carotid artery. Regarding positional symmetry between antimeres, symmetry was observed in 2 (33%) of the specimens, while 4 (67%) showed asymmetry. Among the asymmetric cases, in 3 (75%) the branch on the right antimere emerged more rostrally, and in 1 (25%), the left branch emerged more rostrally. In all females analyzed (100%; 6/6), the caudal cerebral artery was presented as a single vessel between antimeres. In males, the origin of the artery was symmetrical in 4 (80%) specimens, arising from the caudal branch of the internal carotid artery. In the remaining 1 (20%), the right branch originated from the anastomosis between the caudal and rostral branches of the internal carotid artery, whereas the left branch derived exclusively from the caudal branch. Positional symmetry was observed in 3 (60%) of the analyzed male specimens, whereas in the remaining 2 (40%; 2/5), the left branch originated more rostrally. As in females, in all males analyzed (100%; 5/5), the caudal cerebral artery was configured as a single vessel between antimeres.

The mesencephalic artery, in all specimens analyzed, originated at the level of the anastomosis between the caudal branch of the internal carotid artery and the caudal communicating artery. In females, the arrangement was symmetrical between the antimeres in all cases (100%; 6/6) of the specimens examined. Regarding branching patterns, in 5 (83%) of the sample, the mesencephalic artery gave rise to two branches on each antimere, laterally to the cerebral peduncle. In a single specimen, three branches were observed on the right antimere, while the left side maintained the typical two-branch configuration. In males, positional symmetry between the antimeres was also observed in in all cases (100%; 5/5) of the specimens. Regarding branching, in 4 (80%) of the individuals, the mesencephalic artery emitted two branches on each antimere. In 1 (20%) specimen, three branches were observed on the right antimere, a configuration similar to that observed in females.

The rostral branches of the internal carotid artery gave rise to the middle and rostral cerebral arteries along their course on both the right and left antimeres. These branches were interconnected by the rostral communicating artery in all females (100%; 6/6) and in 2 (40%) males. The arterial circle of the encephalon was rostrally open in 60% of the males and caudally closed in all dissected animals. In females, the circle remained rostrally closed by the rostral communicating artery in all cases (100%; 6/6) and caudally closed in the entire sample. In neither sex did the arterial circle exhibit a regular configuration.

The middle cerebral arteries, on both the right and left antimeres, originated from the rostral branch of the internal carotid artery above the optic chiasm in all males and females analyzed. All females exhibited symmetrical emergence in position. Regarding numerical symmetry of the branches, 5 (83%) of the individuals exhibited a symmetrical branching pattern between antimeres, with 2 (40%) showing double branches and 3 (60%) single branches. In the only asymmetry case, four branches were observed on the left antimere and two on the right. In males, the emergence of the artery was symmetrical between antimeres in all cases (100%; 5/5). Regarding the branching pattern, numerical symmetry was observed in 3 (60%) specimens. Among these, two males exhibited two branches on both antimeres, while in one specimen the middle cerebral artery was single bilaterally. In the asymmetric cases, one male presented three branches on the right antimere and two on the left, whereas another exhibited two branches on the left and only one on the right.

In all dissected animals, the rostral cerebral artery originated from the rostral branch of the internal carotid artery, following a straight course along the ventral border of the longitudinal fissure of the encephalon, without fusion at the midline. In females, it exhibited positional symmetry in all cases (100%; 6/6) and remained single in all specimens examined. In males, the rostral cerebral artery also demonstrated positional symmetry between antimeres in 100% of the specimens. In one specimen, a double branch was observed on the left antimere, whereas no branching was identified in the others.

## Discussion

### Basilar artery

No significant difference was observed in the length of the basilar artery between male and female goats studied (p = 0.5305). The reviewed literature did not report corresponding measurements for this vessel in goats. Regarding the sex of the animals, no differences in the length of the basilar artery were observed in crossbred horses (Moraes et al., 2014), Brazilian Shorthair cats (Gomes et al., 2015), and rabbits (Sousa et al., 2016), as also observed in the present study.

In the goats analyzed in the present study, the basilar artery was single in 80% of the males and duplicated in 20%; in females, it was single in 50% and duplicated in 50%. These findings differ partially from those observed in the black-tailed gnu (Zdun et al., 2023), in the moose (Zdun et al., 2019), in the camel (Jerbi et al., 2019), and in the pig (Salaud et al., 2024).

Variations in the basilar artery were described by Souza et al. (2010) in crossbred horses, with variable frequency of the carotidobasilar artery an anastomosis between the internal carotid artery and basilar arteries observed on the ventral surface of the brainstem. This variation was not observed in goats in the present study, nor in the black-tailed gnu (Zdun et al., 2013), moose (Jerbi et al., 2019), species of the genus Bos (Zdun et al., 2013), the scimitar-horned oryx (Zdun et al., 2024), or the buffalo (John et al., 2024).

In both males and females, the basilar artery was formed by the anastomosis of the right and left vertebral arteries, similar to what has been reported in the cat (Lima et al., 2006; Peters et al., 1998; Salaud et al., 2024), the camel (Jerbi et al., 2019), species of the genus Bos (Zdun et al., 2013), the pig (Salaud et al., 2024), and the goat (Alsafy et al., 2019; Salaud et al., 2024; Silva et al., 2025).

The basilar artery gave rise to the following main branches: branches to the bulb, caudal cerebellar arteries, branches to the pons, terminal branches, and rostral cerebellar arteries. This configuration was partially similar to that reported in the black-tailed gnu (Zdun et al., 2013), the moose (Zdun et al., 2019), the camel (Jerbi et al., 2019), the pig (Salaud et al., 2024), the goat (Alsafy et al., 2019; Salaud et al., 2024; Silva et al., 2025), the European fallow deer (Brudnicki, 2011), the scimitar-horned oryx (Zdun et al., 2024), and the buffalo (John et al., 2024).

### Typology

According to the morphological classification proposed by Vriese (1904) for the arteries at the base of the encephalon, the goats analyzed in the present study showed a tendency toward type II and are positioned between the middle and final stages of their phylogenetic development, as considered by Testut (1911). In the same category (type 2), Alcântara and Prada (1996a, 1996b), Lindemann et al. (2000), Lima et al. (2006), and Gomes et al. (2015) classified the vascular arrangement observed respectively in dogs, opossums, and cats.

Regarding the general characteristics of ontogenetic and phylogenetic differentiation of the cerebral arteries, Testut (1911) states that, in primitive conditions, the internal carotid artery is the only artery that supplies blood to the encephalic mass; and the vertebral artery is absent. In the intermediate stage, the two anterior cerebral arteries, which were initially independent, join at the midline, either through an intermediate network or by a simple transverse branch, representing the anterior communicating artery. The two branches of the internal carotid artery, just behind the origin of the future posterior cerebral artery, fuse into a single median trunk, the basilar trunk. In the final stage, the anterior part of the caudal branch of the internal carotid artery undergoes atrophy, and the vertebral artery increases in size, thereby supplying the basilar artery, through which blood then flows from bottom to top. The same occurs with its anterior bifurcation branches (anterior cerebral arteries), which appear to continue this circulation forward.

In the present study, the arterial circle at the base of the brain was rostrally open in 60% of the dissected males and caudally closed in all specimens. In females, the circle was rostrally closed by the rostral communicating artery in 100% of the specimens and caudally closed in the entire sample. Additionally, the contribution of the vertebrobasilar system to the formation of the caudal portion of the encephalic arterial circle positions these specimens between the intermediate and final stages of ontogenetic evolution for this species.

### Formation of the arterial circle at the base of the brain

The arterial circle at the base of the brain shows considerable variation in shape among mammals. In the goats analyzed in the present study, it exhibited an irregular shape. However, according to Alsafy et al. (2019), in goats, the arterial circle has a circular shape. Brudnicki (2000) reported that, in *Capra hircus*, the arterial circle assumed the shape of the number 8 in 23 cases (85.2%), while in the remaining 4 individuals (14.8%) it was heart-shaped.

The shape of the arterial circle at the base of the brain has been described as heart-shaped in the common wildebeest (*Connochaetes taurinus*), the scimitar-horned oryx (*Oryx dammah*), and the water buffalo (*Bubalus bubalis*) (Zdun et al., 2023, 2024; John et al., 2024). In the giraffe (*Giraffa camelopardalis*), the shape is nearly triangular (Frąckowiak & Jakubowski, 2008), whereas in camels (*Camelus dromedarius*), it resembles the number 8 (Kiełtyka et al., 2014).

The vessels of the arterial circle at the base of the brain surround, rostrally, the optic chiasm, the tuber cinereum, and the hypophysis, and caudally, the mammillary bodies and the interpeduncular fossa. In this arrangement, it is possible to observe the rostro-caudal direction of the vessels forming the carotid system and the caudo-rostral direction of the vessels of the vertebrobasilar system, with the anastomosis between the caudal communicating arteries and the terminal branches of the basilar artery representing the convergence point of these two circulatory streams, corroborating the findings reported by Salaud et al. (2024), Silva et al. (2025), Zdun et al. (2023), and Zdun et al. (2024). However, according to Alsafy et al. (2019), in goats, the main sources of blood supply to the brain were the maxillary and basilar arteries.

In the present study, the vessels that defined the arterial circle included: the rostral branch of the internal carotid artery, the rostral cerebral arteries, and the rostral communicating artery; and caudally, the terminal branches of the basilar artery, the caudal branches of the internal carotid artery, and the caudal communicating artery. This configuration was consistent with the findings reported by Alsafy et al. (2019), Salaud et al. (2024), and Silva et al. (2025). Comparable results were also reported by Zdun et al. (2023, 2024).

Silva et al. (2025) observed, rostrally, the closure of the arterial circle at the base of the encephalon by the presence of the rostral communicating artery in 100% of the cases. In contrast, Ashwini et al. (2008) reported that, in some goat specimens, the arterial circle was open rostrally, as also noted by Alsafy et al. (2019). In the present investigation, the arterial circle at the base of the encephalon was rostrally closed in 40% of the males and in 100% of the females, partially agreeing with the findings of Salaud et al. (2024).

In the goat brains analyzed in this study, the arterial circle at the base of the encephalon was caudally closed in all specimens. This morphological arrangement was also reported by Salaud et al. (2024), Silva et al. (2025) and Zdun et al. (2023, 2024).

## Conclusion

The Newborn Saanen goats exhibited variations in the shape and formation of the arterial circle at the base of the brain, with a predominance of the Type II pattern. The basilar artery originated from the anastomosis between the right and left vertebral arteries in all cases, giving rise to encephalic branches consistent with those described in the literature for other species. Rostral closure of the arterial circle was observed in 40% of males and in 100% of females, and caudal closure was present in all specimens. These findings highlight the importance of comparative anatomical studies, especially for understanding brain vascularization in domestic animals and experimental models, providing relevant insights for biomedical research and applications in clinical and surgical practice.
